# Developing BACE-1 inhibitors for FXS

**DOI:** 10.3389/fncel.2013.00077

**Published:** 2013-05-28

**Authors:** Cara J. Westmark, Elizabeth M. Berry-Kravis, Chrysanthy Ikonomidou, Jerry C. P. Yin, Luigi Puglielli

**Affiliations:** ^1^Department of Neurology, University of WisconsinMadison, WI, USA; ^2^Department of Pediatrics, Biochemistry and Neurological Sciences, Rush University Medical CenterChicago, IL, USA; ^3^Department of Genetics, University of WisconsinMadison, WI, USA; ^4^Department of Medicine, University of WisconsinMadison, WI, USA

**Keywords:** fragile X syndrome, BACE-1, Alzheimer’s disease, amyloid-beta, mGluR_5_

## Abstract

Fragile X syndrome (FXS) is a debilitating genetic disorder with no cure and few therapeutic options. Excessive signaling through metabotropic glutamate receptor 5 in FXS leads to increased translation of numerous synaptic proteins and exaggerated long-term depression. Two of the overexpressed proteins are amyloid-beta protein precursor (APP) and its metabolite amyloid-beta, which have been well-studied in Alzheimer’s disease (AD). Here we discus the possibility that pharmaceuticals under study for the modulation of these proteins in AD might be viable therapeutic strategies for FXS. Specifically, a recently identified acetyltransferase inhibitor that reduces the levels and activity of β-site APP cleaving enzyme (BACE-1) has strong potential to attenuate BACE-1 activity and maintain homeostatic levels APP catabolites in FXS.

## INTRODUCTION

Fragile X syndrome (FXS) is the most common form of inherited intellectual disability and the leading known genetic cause of autism (Wang et al., 2010). Clinical features include moderate to severe intellectual disability, autistic-like behavior, anxiety, seizures, and macroorchidism (Hagerman et al., 2009). Neuroanatomical features include an overabundance of long, thin tortuous postsynaptic spines (Beckel-Mitchener and Greenough, 2004). In the majority of cases, FXS is caused by a trinucleotide repeat expansion in the promoter region of the fragile X mental retardation 1 (*FMR1*) gene, which leads to promoter methylation and lack of translation of fragile X mental retardation protein (FMRP). FMRP is an mRNA binding protein that regulates dendritic protein synthesis. Research spanning the past two decades has identified metabotropic glutamate receptor 5 (mGluR_5_) as a key member of a group of Gq-linked receptors that activate dendritic translation through a signaling cascade upstream of FMRP. There has been an intense effort to identify the intermediate signaling molecules in the cascade as well as downstream FMRP mRNA targets (Bhakar et al., 2012). In aggregate, over 500 mRNA ligands have been identified, many with potential to influence synaptic structure and plasticity, but only about a dozen have been validated as evidenced by association with FMRP, dendritic localization, or synaptic synthesis, and regulation by group 1 mGluR (Bassell and Warren, 2008). We validated *App* mRNA as a synaptic target that is translationally regulated by FMRP and mGluR_5_ (Westmark and Malter, 2007). *App* mRNA codes for a transmembrane protein amyloid-beta protein precursor (APP), which is processed by β- and γ-secretases to generate amyloid-beta (Aβ), the predominant protein found in the senile plaques characteristic of Alzheimer’s disease (AD) and Down syndrome. *Fmr1*^KO^ mice, which lack the translational repressor FMRP, exhibit elevated levels of brain APP and Aβ, and the brains of FXS patients also appear to have elevated Aβ (Westmark et al., 2011b). Importantly, downregulation of APP and consequent reduction of Aβ can rescue many phenotypic abnormalities of *Fmr1*^KO^ mice (Westmark et al., 2011b). Thus, it is our opinion that therapies directed at normalizing APP and Aβ levels will benefit FXS. Our opinion is relevant and timely as β-site APP cleaving enzyme (BACE-1) inhibitors are entering clinical trials for the treatment of mild cognitive impairment and AD. Positive results could be rapidly extrapolated to the treatment of FXS, which is considered an orphan disease from the standpoint of treatment development. Herein, we provide a framework for preclinical studies validating APP and Aβ pathophysiology and BACE-1 inhibitor efficacy in animal models of FXS.

## THE mGluR THEORY OF FXS

“The mGluR Theory of Fragile X” proposed by Bear et al. (2004) proposes that overactive signaling by group 1 mGluRs (mGluR_1_ and mGluR_5_) contributes to many of the psychiatric and neurological symptoms of FXS. The theory contends that FMRP binds to synaptic mRNAs and represses their translation. Upon mGluR_5_ activation, FMRP is inactivated or dislodged from target mRNAs, and rapid dendritic synthesis of new proteins leads to long-term depression (LTD) at locally active synapses. In the absence of FMRP, mGluR_5_-mediated translation is constitutive and unregulated. There has been an intense effort by the FXS community to validate the central role of mGluR_5_ in FXS. A 50% genetic reduction of mGluR_5_ levels in *Fmr1*^KO^ mice rescues ocular dominance plasticity, the density of dendritic spines, basal protein synthesis, inhibitory avoidance extinction, audiogenic seizures, and macroorchidism (Dolen et al., 2007); and pharmacological treatment with mGluR_5_ antagonists rescues FXS phenotypes in mouse (*Mus musculus*), fly (*Drosophila melanogaster*), and zebrafish (*Danio rerio*) disease models (McBride et al., 2005; Yan et al., 2005; Tucker et al., 2006; de Vrij et al., 2008; Michalon et al., 2012). In addition, numerous signaling molecules, convergent signaling pathways and other membrane receptors have been identified that contribute to the abnormal synaptic plasticity observed in FXS. Other interacting, and in some cases overlapping, theories have emerged. “The cAMP Theory of FXS” suggests that alterations in cAMP production contribute to FXS neuropathology (Kelley et al., 2007). The “The GABA_A_R Hypothesis” postulates that GABA_A_R is a potential therapeutic target because GABAergic agonists rescue behavioral symptoms of FXS (Heulens et al., 2012). Key FMRP ligands coding for “LTD proteins” have been identified and are potential therapeutic targets (Luscher and Huber, 2010).

## APP AT THE FXS SYNAPSE

We identified *App* mRNA as a synaptic target for mGluR_5_/FMRP regulation (Westmark and Malter, 2007). FMRP binds to a guanine-rich region in the coding region of *App* mRNA and inhibits translation (Westmark and Malter, 2007; Lee et al., 2010). Stimulation with the group 1 mGluR agonist (*S*)-3,5-dihydroxyphenylglycine (DHPG) releases FMRP from the *App* message resulting in increased APP production. In *Fmr1*^KO^ synaptoneurosomes and primary neurons, which lack FMRP, basal APP levels are elevated (Westmark and Malter, 2007; Liao et al., 2008) and do not change in response to DHPG. Consistent with these findings, Aβ levels are elevated in the brain of *Fmr1*^KO^ mice, and several FXS phenotypes including mGluR-LTD can be rescued by genetically reducing APP and Aβ levels (*Fmr1*^KO^/App^HET^ mice; Westmark and Malter, 2007; Westmark et al., 2011b).

Amyloid-beta protein precursor functions in synapse and dendritic spine formation, synaptic transmission, and learning and memory (Hoe et al., 2012). Expression is developmentally regulated with maximal levels during synaptogenesis and subsequent decline when mature connections are completed. Pathological examination of brains from FXS patients shows an increased density of long and tortuous dendritic spines. Similarly, *Fmr1*^KO^ mice exhibit elevated spine protrusion length compared to wild type (WT) littermates. During the first two postnatal weeks, immature filopodia are replaced by mushroom-shaped spines in WT mice whereas *Fmr1*^KO^ exhibit a developmental delay in the transition from immature to mature spines (Cruz-Martin et al., 2010). Likewise, Aβ is strongly implicated in impaired synaptic function (Koffie et al., 2011). Soluble Aβ oligomers facilitate LTD, similar to the enhancement of LTD that occurs in the hippocampus of *Fmr1*^KO^ mice (Huber et al., 2002), and inhibit long-term potentiation (LTP). Aβ is associated with increased hyperexcitability and seizure activity in AD mice (Palop et al., 2007). Interstitial fluid levels of Aβ vary diurnally in both WT and AD mouse models with increased levels associated with wakefulness (Kang et al., 2009). Thus, we predict that the dysregulated expression of APP and its catabolites during FXS development contributes to aberrant synapse formation leading to seizures and behavioral, cognitive and sleep deficits.

**FIGURE 1:**
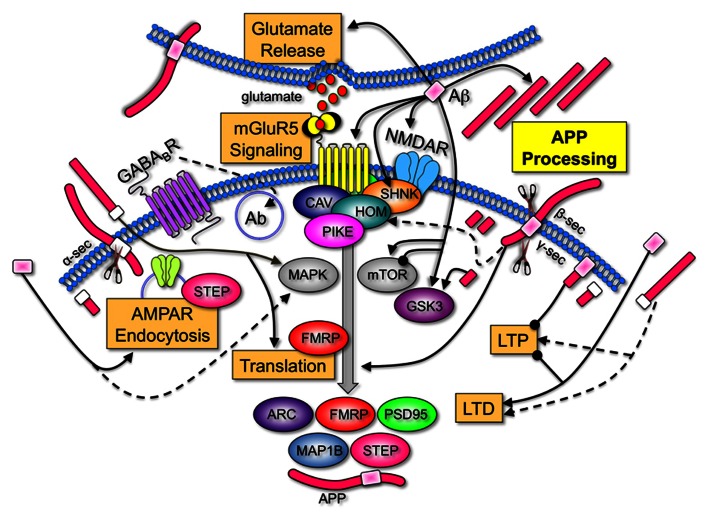
**Amyloid-beta protein precursor and Aβ are key regulators of synaptic activity.** APP is processed by α-, β-, and/or γ-secretases to produce soluble N-terminal domains of APP (sAPPα and sAPPβ), Aβ and C-terminal fragments. Aβ increases LTD, inhibits LTP (Koffie et al., 2011), induces calcium-dependent synaptic vesicle depletion at the presynaptic membrane (Parodi et al., 2010), binds to numerous postsynaptic surface proteins including NMDAR (Danysz and Parsons, 2012), activates mGluR_5_ signaling (Casley et al., 2009; Renner et al., 2010), induces Arc and APP expression (Lacor et al., 2004; Westmark et al., 2011b) and interferes with normal NMDAR and 2-amino-3-(3-hydroxy-5-methyl-isoxazol-4-yl)propanoic acid receptor (AMPAR) trafficking by triggering receptor internalization (Hsieh et al., 2006; Ma and Klann, 2012). Group 1 mGluRs are anchored to NMDAR via a chain of scaffolding proteins including the long isoforms of Homer, Shank, and postsynaptic density protein 95 (PSD95). Aβ induces disassembly of Shank1 and Homer1b clusters (Roselli et al., 2009). Group 1 mGluRs are upstream of two pivotal signaling pathways, PI3 kinase/Akt/mTOR (mammalian target of rapamycin)/p70S6K and Ras/MAPK (mitogen-activated protein kinase)/p90S6K. Both of these pathways regulate the phosphorylation status of eIF4E, 4E-BPs, and ribosomal protein S6, which positively influence protein synthesis. Aβ and sAPP activate MAPK^ERK^ (Young et al., 2009; Chasseigneaux et al., 2011). Aβ both inhibits and activates mTOR signaling (Ma et al., 2010; Caccamo et al., 2011), and activates GSK3β (Takashima et al., 1996), a key regulator of numerous signaling pathways. APP, sAPP, and the intracellular C-terminal fragments also affect synaptic homeostasis. APP anchors cytoplasmic polyadenylation element binding factor (CPEB) to membranes and promotes polyadenylation-induced translation (Cao et al., 2005). sAPPα increases *de novo* protein synthesis (Claasen et al., 2009), enhances LTP (Taylor et al., 2008), shifts the frequency-dependency for induction of LTD (Ishida et al., 1997), and disrupts APP dimers at the plasma membrane (Gralle et al., 2009). While there is only a 17-amino acid difference between the differentially processed N-terminal fragments, sAPPα possesses synaptotrophic and neuroprotective activities while sAPPβ can be toxic (Zheng and Koo, 2011). The C-terminal fragment generated after amyloidogenic processing of APP is also neurotoxic and activates GSK3 (Ryan and Pimplikar, 2005). The 104 amino acid C-terminal fragment containing Aβ impairs LTP (Nalbantoglu et al., 1997). The levels of many synaptic proteins corresponding to a number of FMRP target mRNAs are constitutively elevated in the *Fmr1*^KO^ mouse. A few examples that are regulated by mGluR_5_ are illustrated [ARC (activity-regulated cytoskeleton-associated protein), FMRP, PSD95, MAP1B (microtubule-associated protein 1B), striatal-enriched protein tyrosine phosphatase (STEP), and APP; Bassell and Warren, 2008; Goebel-Goody et al., 2012]. Overall, these data strongly suggest that APP and Aβ are among key LTD proteins whose over-expression during development play an important role in FXS pathogenesis. Key: major cellular processes (glutamate release, mGluR_5_ signaling, AMPAR endocytosis, translation, LTD, LTP, and APP processing) are boxed. Receptors (mGluR_5_, NMDAR, and GABA_B_R) are inserted into the membrane and differentially colored. Scaffolding (Shank, Homer, Caveolin, and Pike), signaling molecules (MAPK, mToR, and GSK3), and translated proteins (ARC, FMRP, PSD95, and MAP1B) are oval-shaped and differentially colored. APP is colored fuchsia with the Aβ portion in light pink for amyloidogenic processing (β- and γ-secretase cleavage) and white for non-amyloidogenic processing (α-secretase cleavage). Secretases are denoted by the scissors symbols. Sharp arrowheads denote activation of a protein or process and rounded arrowheads denote inhibition of a protein or process.

A comparison of the AD and FXS literature demonstrates that many of the identified receptor and signaling molecules with established roles in FXS are regulated by APP and/or Aβ (**Figure 1**). These findings support the contention that APP and Aβ are key LTD proteins that contribute to FXS pathology, and that therapeutics currently under study for the modulation of APP processing and Aβ levels in AD may be applicable to FXS. A caveat to this opinion is that there is no evidence of increased AD pathology in older FXS individuals. Regardless if Aβ forms insoluble plaques in FXS, increasing evidence suggests that soluble, oligomeric forms of Aβ are the pathogenic form of the peptide (Ferreira and Klein, 2011).

## MAINTAINING SYNAPTIC HOMEOSTASIS IN FXS

Deviations in either direction from the optimal level of synaptic proteins can adversely affect plasticity (Kelleher and Bear, 2008). Synaptic deficits in *Tsc2* and *Fmr1* mutant mice are corrected by treatments that modulate mGluR_5_ in opposite directions and disappear in mice that carry both mutations (Auerbach et al., 2011). Similarly, “too much” or “too little” APP and Aβ in *Fmr1*^KO^ mice exacerbates audiogenic seizures (Westmark et al., 2010, 2013). These data support the requirement for maintenance of homeostatic levels of key synaptic proteins in the treatment of FXS and suggest that therapeutic dosages need to be tightly regulated. In fact, it is likely that a cocktail of low dosage drugs will be required to maintain synaptic homeostasis. The results of early-phase clinical trials with targeted FXS therapeutics have been reviewed (Berry-Kravis et al., 2011; Gross et al., 2012). Surprisingly, several of these drugs may be effective in FXS due to off-site activities that modulate APP, Aβ, and/or BACE-1. **Table 1** lists the drugs in clinical trials for FXS, their known activities and their expected effects on APP, Aβ, and BACE-1. Fenobam, lithium, memantine, and minocycline modulate APP, Aβ, and/or BACE-1. The other listed drugs are predicted to modulate these proteins based on their mechanism of action [GABA agonist (Sun et al., 2012), mGluR_5_ antagonist (Westmark and Malter, 2007), glycogen synthase kinase-3 (GSK3) inhibitor (Yu et al., 2012), neurosteroid (Chen et al., 2011), statin (Kojro et al., 2001), serotonin reuptake inhibitor (Cochet et al., 2013), or antioxidants (Heo et al., 2013)].

**Table 1:** Expected effects of drugs in clinical trials for FXS on APP, Aβ, and/or BACE-1.

**Drug (clinical trial sponsor)**	**Drug activity**	**Expected effect**	**Reference**
Acamprosate (Indiana University)	GABA_(A and B)_ agonist	↓ Aβ endocytosis	Erickson et al. (2010)
AFQ056 (Novartis Pharmaceuticals)	mGluR_5_ antagonist	↓ APP and Aβ	Levenga et al. (2011)
Donepezil (Stanford University)	Acetylcholinesterase inhibitor, GSK3 inhibitor	↓ BACE-1 and Aβ	Noh et al. (2009), Sahu et al. (2012)
Fenobam (Neuropharm Ltd; FRAXA)	mGluR_5_ antagonist	↓ APP and Aβ	Berry-Kravis et al. (2009), Malter et al. (2010)
Ganaxolone (Marinus Pharmaceuticals)	Neurosteroid	↓ Aβ	Heulens et al. (2012)
Lithium (FRAXA)	GSK3 inhibitor	↓ BACE-1 and Aβ	Berry-Kravis et al. (2008), Mines and Jope (2011), Yu et al. (2012)
Lovastatin (FRAXA)	Statin	↓ Aβ and ↑ sAPPα	Kojro et al. (2001), Asai et al. (2010), Osterweil et al. (2013)
Memantine (Indiana University)	NMDAR antagonist	↓ APP and Aβ	Erickson et al. (2009), Ray et al. (2010)
Minocycline (UC-Davis; FRAXA)	Antibiotic (tetracycline derivative)	↓ BACE-1, ↑ sAPPα and ↓ Aβ	Paribello et al. (2010), Siopi et al. (2011), Ferretti et al. (2012)
R04917523 (Hoffmann-La Roche)	mGluR_5_ antagonist	↓ APP and Aβ	–
Sertraline (UC-Davis)	Serotonin reuptake inhibitor	↑ ADAM10 and sAPPα	Indah Winarni et al. (2012)
STX107 (Seaside Therapeutics)	mGluR_5_ antagonist	↓ APP and Aβ	–
STX209/Arbaclofen (Seaside Therapeutics)	GABA_(B)_ agonist	↓ Aβ endocytosis	Berry-Kravis et al. (2012)
Vitamins C and E (MIABHR^1^)	Antioxidants	↓ Aβ	–

*^1^The Mediterranean Institute for the Advance of Biotechnology and Health Research*

Amyloid-beta protein precursor and Aβ are implicated in both negative and positive feedback loops predicted to affect synaptic homeostasis. Kamenetz et al. (2003) determined that neuronal activity modulates the generation and secretion of Aβ peptides from hippocampal neurons that overexpress APP. Aβ in turn selectively depresses excitatory synaptic transmission through *N*-methyl D-aspartate receptor (NMDAR) thus completing a negative feedback loop. Renner et al. (2010) showed that Aβ oligomers cause dynamic redistribution of mGluR_5_ to synapses and thus facilitate increased mGluR_5_ signaling. We demonstrated that Aβ induces dendritic APP translation in primary cultured neurons through an mGluR_5_-dependent pathway (Westmark et al., 2011b). Together these studies suggest a positive feedback loop whereby Aβ oligomers facilitate mGluR_5_ signaling leading to increased dendritic APP translation, which provides more target for amyloidogenic processing and the generation of additional Aβ (Ferreira and Klein, 2011; Westmark, 2013).

## SECRETASES MODULATE APP PROCESSING

Anti-Aβ therapies and secretase inhibitors are leading strategies for reducing Aβ in AD. Aβ immunotherapy has proved very effective in reducing soluble Aβ, amyloid plaque and soluble tau as well as associated cognitive decline in AD mouse models; however, there are safety questions (Morgan, 2011). An alternative approach is modulation of secretase activity. β- and γ-secretase inhibitors are currently in the preclinical stage of investigation for AD and could provide a means to reduce amyloidogenic processing in FXS. BACE-1 is a type I transmembrane aspartyl protease that functions as the rate limiting step in the generation of Aβ. A BACE-1 inhibitor significantly reduces plasma and brain Aβ in AD model mice (Ghosh et al., 2008, 2012; Chang et al., 2011). The potential advantage of BACE-1 inhibitors over mGluR_5_ antagonists and anti-Aβ immunotherapy is that the latter therapies can reduce APP and soluble APPalpha (sAPPα) through translational repression and immunodepletion, respectively. APP has normal physiological functions related to synapse formation so it would be advantageous to reduce Aβ while maintaining APP and sAPPα levels. We observed exacerbation of FXS phenotypes in *Fmr1*^KO^ mice treated with a high dose of anti-Aβ or genetically null for APP (*Fmr1*^KO^/App^KO^ mice) suggesting that over-reduction of APP or a catabolite (presumably Aβ) is as toxic as over-expression likely due to the loss of neuroprotective sAPPα. α- and γ-secretases are additional drug targets for reducing Aβ levels. Activation of α-secretases, which cleave within the Aβ transmembrane region, would increase the production of the neuroprotective sAPPα fragment and decrease Aβ. The problem associated with the use of α- or γ-secretase drugs is that they modulate proteolytic processing of other proteins that are critical for cellular function (Vincent and Govitrapong, 2011; Wolfe, 2012). Thus, in our opinion inhibition of BACE-1 is a plausible therapeutic strategy to reduce Aβ and rescue ensuing phenotypes in *Fmr1*^KO^ mice while maintaining APP and sAPPα levels. Unfortunately, the design of BACE-1 inhibitors has proven challenging due to the large size of the catalytic pocket of the enzyme (Gravitz, 2011). As a result, currently identified BACE-1 inhibitors are largely excluded from reaching the central nervous system. BACE-1 inhibitors currently in trials, although able to cross the brain–blood barrier (BBB), display limited brain bioavailability. Therefore, approaches that affect BACE-1 expression levels rather than catalytic activity are being actively sought.

## ER-BASED ACETYLTRANSFERASES REGULATE BACE-1 LEVELS AND ACTIVITY

As part of our AD-related research, we discovered a novel form of post-translational regulation of membrane proteins that has a dramatic impact on BACE-1 metabolism. Specifically, nascent BACE-1 is acetylated in the lumen of the endoplasmic reticulum (ER). The acetylated intermediates are able to reach the Golgi apparatus and complete maturation whereas non-acetylated intermediates are retained in the ER/Golgi intermediate compartment (ERGIC) and degraded (Costantini et al., 2007; Jonas et al., 2008; Pehar and Puglielli, 2013). We identified two novel acetyltransferases (ATases), ATase1 and ATase2, which acetylate BACE-1 and thus regulate its levels and activity (Costantini et al., 2007; Ko and Puglielli, 2009). Both ATases are associated with ER and ERGIC membranes, have one single membrane domain, have a highly conserved catalytic domain that faces the lumen of the organelle (Ko and Puglielli, 2009), are expressed in neurons, and are upregulated in AD brain (Ding et al., 2012). We also identified two novel biochemical compounds, compound 9 (6-chloro-5H-benzo[a]phenoxazin-5-one) and compound 19 (2-chloro-3-(2-ethoxyanilino)-1,4-dihydronaphthalene-1,4-dione), that target ATase-1 and ATase-2 with high specificity and no apparent off-site effects (Ding et al., 2012). In cellular (Ding et al., 2012) and animal models of AD, these compounds dramatically reduce Aβ. Importantly, preliminary studies show that pharmacologic inhibition of ATase1 and ATase2 rescues synaptic deficits and extends the lifespan of APP overexpressing mice without evident toxicity. ATase1 and ATase2 display important structural differences from other classes of ATases (Pehar and Puglielli, 2013), which could explain why widespread off-site effects were not observed when ATase inhibitors were administered to cellular systems (Ding et al., 2012). Importantly, a single-nucleotide polymorphism that inactivates ATase1 has been identified in <2% of the general population. Since no disease association has been reported, we speculate that ATases are viable targets for therapeutic intervention. Thus, the identification of ATase1 and ATase2 has opened a new field of research and sparked interest in manipulating this pathway for therapeutic benefits in AD. While our results were obtained in AD-relevant settings, we propose to extend these findings to FXS.

## BACE-1: A BENCH-TO-BEDSIDE TRANSLATION PLAN FOR FXS

The prospect that FXS phenotypes can be reduced by targeting APP processing is stimulating and deserves close attention. The central hypothesis driving our translation plan is that biochemical inhibition of BACE-1 activity will rescue critical aspects of FXS pathology by reducing amyloidogenic processing of APP. We propose a three-step plan for validating the pathophysiology of APP, Aβ, and BACE-1 in FXS and the efficacy of the ATase inhibitors in attenuating disease phenotypes. Our ultimate goal is to generate the necessary preclinical data for a BACE-1 inhibitor trial in FXS. Inhibition of BACE-1 with ATase inhibitors potentially offers several advantages in the treatment of amyloidogenic disorders including substrate specificity and BBB penetrance.

In Step 1, we propose to assess BACE-1 knockdown in *Fmr1*^KO^ mice on established FXS phenotypes. The creation of tetracycline-inducible *Cre/Fmr1*^KO^/iBACE^HET^ mice would allow for genetic knockdown of BACE-1 at varied points in development (gestational, postnatal, and adult) prior to assessing rescue of phenotypes (seizures, electrophysiology, behavior, sleep, dendritic spine, and biomarker expression). The timing of BACE-1 knockdown with *Cre* technology could provide valuable data regarding the optimal subject age for therapeutic treatments. Chronic pharmacological inhibition of mGluR_5_ reversed established FXS phenotypes in adult *Fmr1*^KO^ mice (Michalon et al., 2012), and a single dose, open-label clinical trial of the mGluR_5_ antagonist fenobam improved prepulse inhibition in adult FXS patients (Berry-Kravis et al., 2009); however, earlier intervention may show improved efficacy. Results with the *Fmr1*^KO^/BACE-1^HET^ mice could then be used as an efficacy standard for pharmacological BACE-1 interventions. Of note, BACE-1 knockdown in *Fmr1*^KO^ mice is expected to reduce Aβ and rescue hyperexcitability and seizures; however, these phenotypes are exacerbated in *BACE-1*^KO^ mice (Hu et al., 2010). Thus, we propose to reduce, not obliterate, BACE-1 activity as some Aβ is required for synaptic homeostasis.

In Step 2, we propose to study the pathophysiology of APP and Aβ in *dfmr* flies. *Drosophila melanogaster *contain both the *dfmr* and *APPL* genes, which are closely related to the mammalian *FMR1* and *APP* genes, and share many of the same disease-related phenotypes. Flies are a less expensive, well-established FXS model (Bushey et al., 2011; McBride et al., 2012; Tessier and Broadie, 2012) and genetic crosses have the potential to elucidate the roles of APP and Aβ in learning, memory, sleep/wake cycles, and biomarker expression.

In Step 3, we propose to inhibit ATase1 with compound 9 in FXS mouse, fly, and human models. Compound 9 efficacy can be compared with BACE-1 knockdown mice, other BACE-1 inhibitors, and anti-Aβ therapies. In addition, the effect of compound 9 on APP processing can be assessed in peripheral blood mononuclear cells (PBMC) isolated from FXS patients. Preliminary studies from our laboratory indicate that Aβ is a potential blood-based biomarker for FXS (Westmark et al., 2011a, b); thus, it is important to understand the effects of BACE-1 inhibitors on both brain and systemic Aβ levels in FXS. Overall, these complementary but distinct approaches to study the biology of APP, Aβ, and BACE-1 in FXS and to rescue disease phenotypes in response to compound 9 could provide solid preclinical data to support testing BACE-1 inhibitors in FXS clinical trials.

## CONCLUDING REMARKS

Due to the inordinate cost of bringing a drug to market, it is highly unlikely that disease-specific drug screens can be performed for more than a couple dozen genetic diseases, leaving the vast majority out of the pharmacological lottery. For these patients and their families, the best hope is repurposing drugs developed for other diseases. Even for single gene diseases with clear etiologies such as FXS, it is unlikely that a single intervention will overcome most of the molecular defects. For example, pharmacological interventions of the well-studied mGluR_5_ pathway in FXS have been successful in overcoming certain (learning deficits and propensity toward audiogenic seizures), but not other (circadian and sleep problems), aspects of the disease in fly and rodent models. This initial research already suggests that “cocktails” of pharmacological treatments will be needed to treat the likely multiple pathways that are affected. From these two perspectives, a cogent case can be made that the identification of “common molecular targets” in different diseases is both economically and scientifically sound. We have identified APP and Aβ as common molecular targets in AD and FXS and we hypothesize that BACE-1 inhibitors, as developed for the treatment of AD, will benefit FXS patients. Herein, we have provided a framework for how APP and Aβ could disturb synaptic homeostasis as well as future directions for generating the necessary preclinical data to justify a pilot clinical trial with a BACE-1 inhibitor in FXS.

## Conflict of Interest Statement

The authors declare that the research was conducted in the absence of any commercial or financial relationships that could be construed as a potential conflict of interest.
